# Minimally invasive versus open McKeown esophagectomy for patients with esophageal squamous cell carcinoma after neoadjuvant PD-1 inhibitor plus chemotherapy

**DOI:** 10.3389/fonc.2023.1103421

**Published:** 2023-01-27

**Authors:** Qiuming Chen, Shaocong Mo, Rusidanmu Aizemaiti, Jun Cheng, Ziheng Wu, Peng Ye

**Affiliations:** ^1^ Department of Thoracic Surgery, The First Affiliated Hospital, College of Medicine, Zhejiang University, Hangzhou, Zhejiang, China; ^2^ Department of Digestive Diseases, Huashan Hospital, Fudan University, Shanghai, China

**Keywords:** esophageal squamous cell carcinoma, neoadjuvant immunochemotherapy, minimally invasive esophagectomy, open surgery, PD-1 inhibitor

## Abstract

**Introduction:**

The purpose of this study was to compare short and mid-term outcomes in esophageal squamous cell carcinoma (ESCC) patients undergoing open or minimally invasive McKeown esophagectomy (MIE) after neoadjuvant PD-1 inhibitor plus chemotherapy.

**Methods:**

Patients with locally advanced ESCC underwent open or minimally invasive McKeown esophagectomy after neoadjuvant PD-1 inhibitor plus chemotherapy were retrospectively included from June 2019 to June 2021. The baseline characteristics, pathological data, short-and mid-term outcomes were collected and compared based on the surgical approach.

**Results:**

A total of 35 patients were included in the study. An open procedure was performed for 13 patients (37.1%), and 22 (62.9%) patients underwent MIE after neoadjuvant therapy. Compared with open group, MIE group had shorter operative times (350.8± 117.8 *vs*. 277.9 ± 30.2 min, P = 0.009). The total number of resected lymph nodes was not significantly different, but more left recurrent laryngeal lymph nodes were harvested from the Open group (2.6 ± 3.2 *vs*. 0.9 ± 1.7, P = 0.047). The median follow-up time was 1.42 years (range, 0.35–2.59 years) from the first day of treatment. Three patients (8.6%) died during follow-up, one in the open surgery group and two in the MIE group. There were six (17.1%) patients developed recurrence, three in each group. The 2-year cumulative survival rates were 92.3 ± 7.4% and 89.5 ± 7.1% for the open and MIE groups, respectively. Overall survival was not different between the two surgical approaches.

**Conclusions:**

MIE might be safe and feasible for patients with locally advanced ESCC undergoing neoadjuvant PD-1 inhibitor plus chemotherapy.

## 1 Introduction

Esophageal cancer is the seventh most common cancer and the sixth leading cause of cancer-related deaths globally in 2020 (1). More than half of the world’s esophageal cancer cases occur in China. Esophageal squamous cell carcinoma (ESCC) is the main sub-type in China, accounting for 90% of all esophageal cancers. Most patients are diagnosed with locally advanced disease because the early stage of ESCC is usually asymptomatic. Surgery remains the primary treatment option. However, surgery alone is often associated with high recurrence and metastasis rates in patients with locally advanced esophageal cancer. Therefore, neoadjuvant chemoradiotherapy or chemotherapy followed by surgery has been gradually adopted as the first choice for patients with resectable, locally advanced ESCC on ground of a series of multi-institutional clinical trials (2, 3). However, the 5-year OS rate remains far from satisfactory and half of patients developed recurrence within 5 years postoperatively (4). Therefore, developing novel efficacious therapeutic strategies is urgently needed to improve clinical outcomes of patients with locally advanced ESCC.

In recent years, the inhibition of programmed death receptor 1 (PD-1) and its ligand combined with chemotherapy have demonstrated great promising benefits for patients with advanced ESCC (5). Camrelizumab (SHR-1210, Jiangsu Hengrui Pharmaceuticals Co, Ltd), a humanized, selective IgG4-k monoclonal antibody against PD-1, showed antitumor activity in multiple solid tumors (6). The phase III ESCORT-1st study confirmed that the first-line camrelizumab plus chemotherapy significantly improved survival in patients with untreated advanced ESCC (7). Immunotherapy plus chemotherapy has been exploratory application in the neoadjuvant setting and several clinical trials had also been reported. However, data on the safety and efficacy of neoadjuvant camrelizumab combined with chemotherapy are limited (8, 9). In addition, chemotherapy induced tissue edema and adhesion increase the difficulty of the dissection of the primary tumor and lymph nodes, which increasing the accidental injury of adjacent structures. Despite its potential benefits, questions remain regarding the safety of minimally invasive McKeown esophagectomy (MIE) for ESCC patients receiving PD-1 inhibitors plus chemotherapy, as it may make the procedure more technically demanding. Therefore, the more appropriate surgical procedure is still indefinite for ESCC patients received neoadjuvant PD-1 inhibitor plus chemotherapy.

This retrospective study aimed to compare short- and mid-term clinical outcomes between MIE and traditional open surgery for locally advanced ESCC patients who received neoadjuvant PD-1 inhibitor plus chemotherapy.

## 2 Patients and methods

Ethical statement: This retrospective study was approved by the Institutional Review Board of The First Affiliated Hospital of Zhejiang University School of Medicine (Ethics approval No.: 2020-550). Written patients informed consent was waived by the Institutional Review Board.

A retrospective data analysis was undertaken on all patients with locally advanced ESCC who received neoadjuvant PD-1 inhibitors plus chemotherapy followed by open or minimally invasive McKeown esophagectomy at the First Affiliated Hospital of Zhejiang University School of Medicine between June 2019 to June 2021. There was no commercial support for the study. The study was designed and written by the authors, who ensured the accuracy and completeness of the reported data and compliance with the study protocol. All tumors met the following criteria: (1) histologically confirmed ESCC; (2) potentially resectable and locally advanced ESCC, defined as cT1N1-3M0 or cT2-4aN0-3M0 [Union for International Cancer Control TNM Classification, 8th Edition (10)]; (3) located in the thoracic esophagus. Eligible patients are 18 to 75 years old with Eastern Cooperative Oncology Group (ECOG) ≤2. Patients were excluded if they had an immunodeficiency disease or were undergoing immunosuppressive therapy with either corticosteroids or other immunosuppressive drugs within the previous 2 weeks or had a history of the use of anti-PD-1 or anti-PD-L1 medication, paclitaxel, or carboplatin.

### 2.1 Neoadjuvant treatment

Patients received at least two cycles of neoadjuvant treatment before surgery. The detailed treatment regimen was as follows: (1) an intravenous PD-1 inhibitor (200 mg of camrelizumab) and nanoparticle albumin-bound paclitaxel (260 mg/m^2^) on day 1 and (2) carboplatin (area under the curve, 5; 5 mg/mL/min) or cisplatin (60 mg/m^2^) on day 2 during each 21-day cycle. After two cycles of neoadjuvant treatment, contrast-enhanced thoracoabdominal computed tomography was performed to assess the treatment response according to the Response Evaluation Criteria in Solid Tumors, version 1.1. A multidisciplinary discussion determined whether to continue with neoadjuvant therapy or perform surgery.

### 2.2 Surgery

About 3-6 weeks after neoadjuvant therapy, open or minimally invasive Mckeown esophagectomy was performed for the patients. The decision for MIE or open surgery depends on the surgeon’s inclination. Briefly, open Mckeown esophagectomy involved a right posterolateral thoracotomy in the lateral decubitus position, upper midline laparotomy, and cervical anastomosis. MIE was firstly performed through a right thoracoscopy in the left lateral decubitus position with four thoracoscopic ports. The thoracic esophagus is mobilized from the thoracic inlet to the diaphragmatic reflection with dissection of the recurrent laryngeal nerve, paraesophageal and subcarinal lymph nodes. After closing the thoracic ports, the patient would be turned to the supine position to perform the laparoscopic procedure with gastric mobilization and upper abdominal lymph node dissection, followed by reconstruction of the neo-esophagus and performance of neck anastomosis, which was the same with open surgery. Two-field lymphadenectomy was routinely performed in both procedures.

### 2.3 Assessment

After surgery, pathological examination of the resected specimens was performed to evaluate the resection margin status and the tumor regression grade (TRG) (11, 12). Pathological complete response (PCR) was defined as the absence of viable tumor cells in the resected cancer specimen. Major pathological response (MPR) was defined as less than 10% of residual viable tumor cells. R0 resection was defined as a microscopically margin-negative resection.

All patients were recommended to reexamination regularly after being discharge from the hospital. Patients were followed up every 3 months during the first year after surgery and every 6 months in subsequent years. Overall survival (OS) was defined as the time from the date of first neoadjuvant treatment to death by any cause. Disease-free survival (DFS) was defined as the time from the date of first neoadjuvant treatment to recurrence. The primary endpoints were safety and feasibility, and the secondary endpoints included overall survival and disease-free survival.

### 2.4 Statistical analysis

The categorical variables were presented as frequencies and percentages. Continuous variables were expressed with a mean ± standard deviation when the normality was verified by Shapiron-Wilk test (P > 0.1), otherwise median and range. Spearman’s correlation was used to assess associations. Comparisons between the subgroups were performed using chi-square tests or Fisher’s exact test. The median follow-up time was calculated by using the Kaplan–Meier method. The Kaplan–Meier method was used to calculate DFS and OS. The reported P values are bilateral, and the significance level was set at 0.05 for all analyses unless otherwise indicated.

## 3 Results

### 3.1 Patient characteristics

Between June 2019 to June 2021, a total of 35 patients with locally advanced ESCC were included in this study. All the patients underwent neoadjuvant camrelizumab plus chemotherapy followed by McKeown esophagectomy, and patients were grouped by the surgical procedure. The cohort was primarily male (n = 29, 82.9%) with a median age of 65 years (range, 46–78 years; **Table 1**), 40% with smoking history, 60.0% with drinking history, and 25 (71.4%) without other medical condition. Besides, 57.1% of the tumors located in the middle segment of the esophagus, and 17 (48.6%) patients were diagnosed with stage III ESCC. The detailed clinical characteristics of the two groups are summarized in **Table 1**.

**Table 1:** Baseline characteristics of patients stratified by the surgical approach.

Demographics	Open surgery (N=13)	MIE (N=22)	*P*
Age (y)	64.0 ± 7.8	67.0 ± 6.7	0.237
Gender			
Female	2 (15.4%)	4 (18.2%)	0.832
Male	11 (84.6%)	18 (81.8%)	
Location			0.378
Upper third	0 (0)	3 (13.6%)	
Middle third	8 (61.5%)	12 (54.5%)	
Lower third	5 (38.5%)	7 (31.8%)	
Pretherapeutic clinical stage			0.789
I	0	0	
II	1 (7.7%)	3 (13.6%)	
III	6 (46.2%)	11 (50.0%)	
IV	6 (46.2%)	8 (36.4%)	
Neoadjuvant therapy cycles			1.000
2	10 (76.9%)	17 (77.2%)	
3	3 (23.1%)	5 (22.7%)	

MIE, minimally invasive McKeown esophagectomy.

### 3.2 Neoadjuvant treatment and outcome

All patients completed neoadjuvant camrelizumab plus chemotherapy. No patients withdrew from neoadjuvant therapy due to toxic effects. Twenty-seven (77.1%) patients received two cycles of the neoadjuvant treatment, and the rest of eight patients received three cycles. Cisplatin was used in 28 patients (80.0%). Treatment-related AEs were manageable and summarized in **Table 2**. Hematological AEs included anemia, a decreased white blood cell count, a decreased neutrophil count, and thrombocytopenia were experienced by 28 (80.0%), 14 (40.0%), 11 (31.4%), and 4 (12.5%) patients, respectively. The most common grade 3 AE was anemia (2, 5.7%). Thirteen (37.1%) patients recorded grade 1 or 2 reactive cutaneous capillary endothelial proliferation, which is commonly associated with camrelizumab. Common toxicities associated with immunotherapy, such as pneumonitis, myocarditis and hypophysitis, were not observed.

**Table 2:** Adverse events during neoadjuvant therapy.

Variables	Any grade	Grades 1–2	Grade 3	Grade 4-5
Low white blood cell count	14 (40.0%)	14 (40.0%)	0	0
Low neutrophil count	11(31.4%)	11 (31.4%)	0	0
Low lymphocyte count	29 (82.9%)	25 (71.4%)	4 (11.4%)	0
Anemia	28 (80.0%)	26 (74.3%)	2 (5.7%)	0
Low platelet count	4 (11.4%)	4 (11.4%)	0	0
Alanine aminotransferase increased	3 (8.6%)	3 (8.6%)	0	0
Aspartate aminotransferase increased	1 (2.9%)	1 (2.9%)	0	0
Reactive cutaneous capillary endothelial proliferation	13 (37.1%)	13 (37.1%)	0	0
Nausea or vomiting	8 (22.9%)	0	0	0
Decreased appetite	6 (17.1%)	0	0	0
Fatigue	12 (34.2%)	0	0	0
Rash	5 (14.3%)	5 (14.3%)	0	0
Myocarditis	0	0	0	0
Hepatitis	0	0	0	0
Pneumonia	0	0	0	0
Hyperthyroidism	0	0	0	0

Based on radiological evaluation after neoadjuvant therapy, all 35 patients showed a reduction in tumor size. Two (5.7%) patients achieved a radiological CR, 26 (74.3%) patients achieved a radiological PR, and 7 (20.0%) patients had varying degrees of reduction, but did not meet the PR criteria. None of the patients showed disease progression during neoadjuvant therapy. Accordingly, the clinical overall objective response rate (ORR) and disease control rate (DCR) was 80.0% and 100%, respectively.

Postoperative pathologic analysis showed that 4 (11.4%) patients achieved PCR and six (17.1%) patients achieved MPR. In addition, 28 (80.0%)patients obtained preoperative clinical downstaging, and 22 (62.9%) achieved postoperative pathological downstaging. Thirteen of 32 (40.6%) patients with node positivity before neoadjuvant therapy achieved nodal clearance. Postoperative pathological analysis showed that 14 patients (40.0%) were stage IA to IIB, and 21 patients (60.0%) were stage IIIA to IVA. No difference was found between the two surgical approaches about Pathological stage after neoadjuvant treatment, which was showed in the **Table 3**.

**Table 3:** Pathological stage after neoadjuvant treatment of patients stratified by the surgical approach.

Demographics	Open surgery (N=13)	MIE (N=22)	*P*
**Neoadjuvant therapy cycles**			1.000
2	10 (76.9%)	17 (77.2%)	
3	3 (23.1%)	5 (22.7%)	
**Pathological T stage**			0.347
0	0	4 (18.2%)	
T1	3 (21.4%)	5 (22.7%)	
T2	4 (28.6%)	6 (27.3%)	
T3	6 (42.9%)	7 (31.8%)	
**Pathological N stage**			0.309
N0	4 (30.8%)	12 (54.5%)	
N1	6 (46.2%)	6 (27.3%)	
N2	2 (15.4%)	4 (18.2%)	
N3	1 (7.7%)	0 (0)	
**ypTNM stage**			0.445
I	3 (23.1%)	9 (40.9%)	
II	1 (7.7%)	2 (9.1%)	
III	8 (61.6%)	11 (50.0%)	
IV	1 (7.7%)	0 (0)	
Downstaging of T stage	8 (61.5%)	15 (68.2%)	0.726
Downstaging of N stage	6 (46.2%)	14 (63.6%)	0.481
Downstaging of TNM stage	8 (61.5%)	4 (63.6%)	1.000
**PCR**	0	4 (18.2%)	0.274
**MPR**	2 (15.4%)	4 (18.2%)	1.000

PCR, pathological complete response; MPR, major pathological response; MIE, minimally invasive McKeown esophagectomy; ypTNM, neoadjuvant pathologic stage TNM.

### 3.3 Surgical treatment and outcome

All patients underwent scheduled Mckeown esophagectomy, R0 resection was achieved in all patients. No treatment-related surgical delays were recorded, and the mean interval from the end of neoadjuvant therapy to surgery was 32.4 ± 3.9 days (Range: 23–40). Thirteen (37.1%) patients underwent open surgery, while 22 (62.9%) patients underwent MIE. No patient converted to open surgery. Compared with patients who underwent open surgery, patients who underwent MIE had a shorter operative time (350.8± 117.8 *vs*. 277.9 ± 30.2min, P = 0.009). The total number of resected lymph nodes was not significantly different between the two groups, but more left recurrent laryngeal lymph nodes were harvested from the open surgery group than the MIE group (2.6 ± 3.23 *vs* 0.91 ± 1.7; P = 0.047).

The postoperative complications are summarized in **Table 4**. No patients died in the hospital. Sixteen patients (45.7%) developed postoperative complications. The most common postoperative complication was left pleural effusion requiring drainage (31.4%). One patient in the MIE group underwent reoperation because of uncontrolled chylothorax. Anastomotic leakage occurred in two patients during the hospital stay, with one in each group (Open group: 7.7% *vs*. MIE group: 4.5%, P = 1.00). The length of postoperative hospital stay was not significantly different between the two groups, but the open surgery group had more patients with postoperative hospital stays of more than 2 weeks (38.5% *vs*. 27.3%, P = 0.708).

**Table 4:** Comparison of postoperative outcomes between open surgery and minimally invasive approaches.

Variables	Open surgery (N=13)	MIE (N=22)	*P*
R0 resection	13 (100%)	22 (100%)	1.000
Operative time (min)	350.8 ± 117.8	277.9 ± 30.2	**0.009**
Total resected lymph nodes	29.7 ± 12.0	27.4 ± 11.7	0.583
Left recurrent laryngeal lymph nodes	2.6 ± 3.2	0.9 ± 1.7	**0.047**
Right recurrent laryngeal lymph nodes	3.1 ± 2.0	2.9 ± 2.8	0.849
Pathological complete response	0	4 (18.2%)	0.274
Postoperative hospital stays (d)	15.0 ± 8.5	15.5 ± 10.4	0.884
Postoperative hospital stays > 2 w	5 (38.5%)	6 (27.3%)	0.708
In-hospital mortality, n (%)	0 (0)	0 (0)	1.000
In-hospital complications			
Anastomotic leakage	1 (7.7%)	1 (4.5%)	1.000
ARDS	0	2 (9.1%)	0.519
Chylothorax	1 (7.7%)	1 (4.5%)	1.000
Arrhythmia	0	1 (4.5%)	1.000
Recurrent laryngeal nerve injury	1 (7.7%)	1 (4.5%)	1.000
Mortality after discharge	1 (7.7%)	2 (9.1%)	1.000
Anastomotic stricture	1 (7.7%)	1 (4.5%)	1.000
Recurrence, n (%)	3 (23.1%)	3 (13.6%)	0.647

MIE, minimally invasive McKeown esophagectomy; ARDS, acute respiratory distress syndrome.

### 3.4 Follow-up

**Figure 1:**
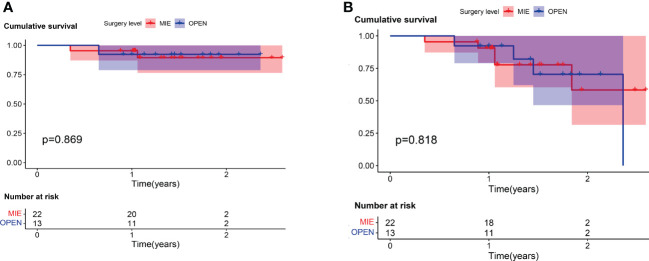
For patients who received camrelizumab plus chemotherapy, no significant difference was found in **(A)** overall survival (log rank = 0.027; *P* = 0.869) or **(B)** disease-free survival (log rank = 0.156; *P* = 0.693) between patients who underwent open surgery (Open) and those who underwent minimally invasive (MIE) procedures.

The median follow-up time was 1.42 years (range, 0.35–2.59 years) from the first day of neoadjuvant treatment. Three patients (8.6%) died during follow-up, one in the open surgery group and two in the MIE group. One patient with cT2N0M0 stage disease in the open surgery group died suddenly 6 months after surgery. In the MIE group, patient one with cT4aN3M0 stage who achieved an MPR after two cycles, died for drug-induced liver injury 10 months after surgery. Another patient with cT3N1M0 stage died for deep venous thrombosis 2 months after surgery. There were six (17.1%) patients developed recurrence during follow-up, three in the open surgery group and three in the MIE group. Four patients developed local recurrence in the regional lymph nodes. Two patients developed the distant metastasis, one with supraclavicular lymph nodes metastasis in the open group, the other with pulmonary metastasis and mediastinal lymph node metastasis in the MIE group. There was no significant difference between the two groups about the recurrence site. The mean DFS time was 1.46 ± 0.49 years, with a 2-year DFS rate of 81.8%. Meanwhile, the mean OS time was 1.45 ± 0.49 years, with a 2-year OS rate of 90.7%. The 2-year cumulative survival rates were 92.3 ± 7.4% and 89.5 ± 7.1% for the open surgery and MIE groups, respectively (**Figure 1**). Kaplan-Meier analyses suggested that patients underwent the two surgical approaches had no significant differences in OS and in DFS.

## 4 Discussion

To the best of our knowledge, this is the first study to compare the outcomes of the open surgery and MIE approaches in ESCC patients after neoadjuvant immunochemotherapy. The results of this study showed that (1) after neoadjuvant therapy of camrelizumab plus chemotherapy, the clinical ORR and DCR were 80.0% and 100%, respectively; (2) after surgery, the PCR and R0 resection rates were 11.4% and 100%, respectively; (3) the 2-year cumulative survival rates were 92.3 ± 7.4% and 89.5 ± 7.1% for the open and MIE groups, respectively. Accordingly, MIE might be safe and feasible for ESCC patients who received neoadjuvant camrelizumab plus chemotherapy.

ESCC remains a challenging malignancy with a poor prognosis and limited therapeutic options. Although improvements in surgical techniques and neoadjuvant therapy strategies for locally advanced ESCC have been achieved, the survival rates for ESCC patients after multimodal therapy remain unsatisfactory. Although neoadjuvant chemoradiotherapy can further increase the R0 resection rate, it is associated with more postoperative complications and higher postoperative mortality. Developing novel efficacious therapeutic strategies is urgently needed to improve prognosis. Immunotherapy has recently been highlighted for the treatment of solid tumors. Several trials are currently underway to assess neoadjuvant anti-PD-1/PDL1 therapy combined with chemotherapy for locally advanced ESCC (13, 14). Here, we showed that neoadjuvant camrelizumab plus chemotherapy had favorable safety and feasibility. The incidence of AEs was lower in this study than in previous studies (15–17). There were no serious immune-related AEs, which may be related to the better physical condition of our patients compared with patients with advanced or metastatic disease. Therefore, neoadjuvant camrelizumab therapy does not increase the incidence of side effects when combined with chemotherapy for ESCC patients.

Although neoadjuvant chemotherapy contributes to tumor downstaging to allow a more radical surgical resection, it may result in tissue edema and adhesion, which makes tumor dissection more difficult and increases the likelihood of bleeding and injury to adjacent organs. Technical challenges and perioperative issues are concerns for patients after neoadjuvant immunotherapy with chemotherapy. Open esophagectomy appeared to have advantages in dealing with tissue adhesion and bleeding under direct vision. With the development of endoscopic techniques, MIE is gaining popularity for the treatment of ESCC, as it is less invasive and has lower complication rates (18, 19). Previous studies have shown that MIE is a safe and acceptable surgical technique for locally advanced esophageal cancer after neoadjuvant therapy (20, 21). There are very limited data on the short- and long-term clinical outcomes of open esophagectomy or MIE for ESCC patients after neoadjuvant PD-1 inhibitor plus chemotherapy (18, 22). Therefore, it is essential to determine the appropriate surgical method for esophagectomy after neoadjuvant PD-1 inhibitor plus chemotherapy.

In this study, patients with locally advanced ESCC receiving neoadjuvant camrelizumab plus chemotherapy reached a clinical ORR of 80.0% and a DCR of 100%. This therapeutic strategy conferred a PCR and MPR in 28.6% of resected tumors. The PCR rates with the doublet chemotherapy drugs were approximately 2.5–33% in previous studies (3, 23). Shen et al. reported an R0 resection rate of 96.3% and a PCR rate of 33.3% in patients with locally advanced ESCC receiving neoadjuvant nivolumab or pembrolizumab plus chemotherapy (15). In the current study, 4(11.4%) patients achieved a PCR, which was much lower than the rates reported in previous studies (15–17). This may be explained by the fact that 40% of our cohort was at clinical stage IVa, and most of them received only two cycles of neoadjuvant treatment. It is possible that an increased number of cycles may improve the treatment effect and achieve a higher PCR rate, but it may also increase toxicity and side effects.

The neoadjuvant therapy in this study did not delay surgery, and the R0 resection rate reached 100%, while in previous studies, the reported R0 resection rates with neoadjuvant chemotherapy and neoadjuvant chemoradiotherapy were 60% and 98.4%, respectively (2, 24). We found that a shorter operation time could be achieved in the MIE group, which was consistent with the findings of previous studies (21). There was no conversion to open surgery. The postoperative complications were similar between our study and previously reported studies, which indicated that neoadjuvant immunotherapy treatment did not increase the risks or complications associated with surgery (15, 17). With regard to the concern on tissue edema and adhesion attributed to neoadjuvant chemotherapy, we found that most of the esophageal tumors more loosely adhered to the surrounding tissues after neoadjuvant PD-1 inhibitor plus chemotherapy, which was in line with the report by Chen and colleagues (21). The quality of esophagectomy was not decreased by neoadjuvant camrelizumab combined with chemotherapy. The reason may be that camrelizumab and chemotherapy worked synergistically to yield a favorable therapeutic response, which needs to be verified in future study.

Lymph node metastasis is closely associated with poor prognosis in ESCC. Therefore, the dissection of lymph nodes was very important in the esophagectomy, especially along with the recurrent laryngeal nerve nodes. The average number of resected lymph nodes (29.6) was significantly higher than the numbers reported in the CROSS (15.0) and NEOCRTEC5010 (20.0) studies (25), which were similar with the number reported by Yang et al. (17) This indicated that the quality of lymph node dissection was not decreased when immunotherapy was added to chemotherapy. This may be due to immunotherapy itself, prompting an immune response and thus leading to better lymph node yields. There was no significant difference in the number of dissected lymph nodes between the two groups in this study, which was similar to previously reported results. Our study confirmed that dissection along the recurrent laryngeal nerve was feasible and safe. However, in contrast to our initial assumption, fewer lymph nodes were harvested along the left recurrent laryngeal nerve in MIE group than Open group. Maybe it’s more difficult to perform the systematic lymphadenectomy along with the recurrent laryngeal nerve in MIE. Similar with our study, Chen et al. from Sun Yat-sen University Cancer Center also found the lymph node yield in open surgery group was higher than in MIE group (21). The reason may be that the traditional open surgery is beneficial to perform the systematic lymphadenectomy under direct vision, while the operation field observed by the monitor in MIE was lacking in partial depth perception due to its two dimensions (21).

This study has several inherent limitations. First, it was limited by its retrospective design which may cause biases. Patients were not randomly assigned to open esophagectomy or MIE group but were treated based on surgeon’s inclination. Second, the number of included patients was small. Third, the follow-up time was short. Long-term follow-up is necessary to assess the effect of neoadjuvant camrelizumab plus chemotherapy on disease-free and overall survival.

In conclusion, MIE might be safe and feasible for patients with locally advanced ESCC who undergoing neoadjuvant camrelizumab plus chemotherapy in this small, retrospective study. The optimal treatment regimen and long-term results are important issues that need further investigation.

## Data availability statement

The original contributions presented in the study are included in the article/**Supplementary Material**. Further inquiries can be directed to the corresponding author.

## Ethics statement

This retrospective study was approved by the Institutional Review Board of The First Affiliated Hospital of Zhejiang University School of Medicine (Ethics approval no: 2020-550). The ethics committee waived the requirement of written informed consent for participation.

## Author contributions

All authors made substantial contributions to the conception of the study. QC, SM and PY conceptualized the study, revised the manuscript and supervised the study. QC and ZW collected the data, drafted the manuscript and made the figures RA, JC and PY revised the manuscript. All authors contributed to the article and approved the submitted version.
