# Supplementary material for: Minimally invasive versus open McKeown esophagectomy for patients with esophageal squamous cell carcinoma after neoadjuvant PD-1 inhibitor plus chemotherapy
Source: Front Oncol. 2023 Jan 27;13:1103421. doi: 10.3389/fonc.2023.1103421 (PMC9912456; doi:10.3389/fonc.2023.1103421)
Supplement: Supplementary file 1 [file Table_1.docx]

| **patient** | sex | age | MIE | cT | cN | cTNM | ypT | ypN | ypStage |
| --- | --- | --- | --- | --- | --- | --- | --- | --- | --- |
| **1** | 1 | 70 | 1 | 3 | 1 | III | 2 | 0 | I |
| **2** | 1 | 54 | 1 | 3 | 3 | IVA | 0 | 0 | 0 |
| **3** | 2 | 73 | 1 | 4 | 3 | IVA | 1 | 0 | I |
| **4** | 1 | 68 | 0 | 2 | 0 | II | 2 | 0 | I |
| **5** | 1 | 74 | 0 | 3 | 1 | III | 2 | 1 | IIIA |
| **6** | 1 | 70 | 1 | 3 | 2 | III | 0 | 0 | 0 |
| **7** | 1 | 72 | 0 | 3 | 1 | III | 3 | 1 | IIIB |
| **8** | 1 | 55 | 1 | 3 | 3 | IVA | 1 | 2 | IIIB |
| **9** | 1 | 69 | 1 | 3 | 1 | III | 3 | 1 | IIIA |
| **10** | 2 | 71 | 0 | 4 | 1 | IVA | 1 | 1 | IIIA |
| **11** | 1 | 69 | 1 | 3 | 1 | III | 3 | 1 | IIIB |
| **12** | 1 | 75 | 1 | 3 | 3 | IVA | 3 | 2 | IIIB |
| **13** | 1 | 69 | 1 | 3 | 1 | III | 3 | 1 | IIIB |
| **14** | 1 | 63 | 0 | 3 | 3 | IVA | 1 | 0 | I |
| **15** | 1 | 56 | 0 | 3 | 3 | IVA | 1 | 1 | IIIA |
| **16** | 2 | 78 | 1 | 3 | 2 | III | 0 | 0 | 0 |
| **17** | 1 | 64 | 0 | 4 | 3 | IVA | 3 | 3 | IVA |
| **18** | 1 | 65 | 0 | 3 | 3 | IVA | 2 | 2 | IIIA |
| **19** | 2 | 65 | 0 | 3 | 1 | III | 3 | 1 | IIIA |
| **20** | 1 | 46 | 0 | 3 | 2 | III | 3 | 2 | IIIB |
| **21** | 1 | 72 | 1 | 4 | 3 | IVA | 1 | 2 | IIIB |
| **22** | 2 | 68 | 1 | 3 | 2 | III | 2 | 1 | IIIA |
| **23** | 1 | 65 | 1 | 4 | 1 | IVA | 2 | 0 | IIIA |
| **24** | 1 | 60 | 1 | 3 | 2 | III | 3 | 2 | IIIB |
| **25** | 1 | 59 | 1 | 4 | 3 | IVA | 2 | 0 | I |
| **26** | 1 | 63 | 1 | 2 | 1 | II | 1 | 1 | IIIA |
| **27** | 1 | 68 | 1 | 3 | 1 | III | 0 | 0 | 0 |
| **28** | 1 | 55 | 0 | 4 | 2 | IVA | 3 | 1 | IIIB |
| **29** | 1 | 55 | 1 | 3 | 3 | IVA | 2 | 0 | I |
| **30** | 1 | 73 | 1 | 3 | 0 | II | 1 | 0 | I |
| **31** | 1 | 66 | 1 | 3 | 1 | III | 3 | 0 | II |
| **32** | 2 | 72 | 1 | 3 | 0 | II | 3 | 0 | II |
| **33** | 1 | 64 | 0 | 3 | 1 | III | 3 | 0 | II |
| **34** | 1 | 69 | 0 | 3 | 1 | III | 2 | 0 | I |
| **35** | 1 | 71 | 1 | 3 | 1 | III | 2 | 1 | IIIA |
